# Selective Androgen Receptor Modulators (SARMs)-Induced Liver Injury: A Case Report and Review of Literature

**DOI:** 10.7759/cureus.35094

**Published:** 2023-02-17

**Authors:** Wael T Mohamed, Vinay Jahagirdar, Ifrah Fatima, Mohamed K Ahmed, Fouad Jaber, Kevin Wang, Amira Hassan, Eric Ewing, Wendell Clarkston, Alisa Likhitsup

**Affiliations:** 1 Internal Medicine, University of Missouri Kansas City, Kansas City, USA; 2 Pathology, University of Missouri Kansas City, Kansas City, USA; 3 Gastroenterology and Hepatology, University of Missouri Kansas City, Kansas City, USA

**Keywords:** drug-induced hepatitis, hepatotoxic agent, hepatotoxic drugs, drug-induced liver injury (dili), selective androgen receptor modulators

## Abstract

Drug-induced liver injury (DILI) is one of the leading causes of death from acute liver failure (ALF) in the United States, accounting for approximately 13% of ALF cases in the United States. Selective androgen receptor modulators (SARMs) were first developed to increase muscle mass while avoiding the side effects of conventional androgenic steroids. Although not Food and Drug Administration (FDA) approved, they are widely available online and are consumed to enhance athletic performance. We report a 22-year-old, previously healthy male, who presented with a two-week history of worsening jaundice, nausea, fatigue, pruritus, dark urine, and light stools. He reported taking the SARM, RAD-140, for 16 weeks. Examination showed scleral icterus. The liver panel showed alkaline phosphatase (ALP) 5.3 µkat/L, alanine transaminase (ALT) 1.66 µkat/L, aspartate transaminase (AST) 1.18 µkat/L, direct bilirubin 294 µmol/L, total bilirubin 427.5 µmol/L, and international normalized ratio (INR) 0.9. Viral hepatitis and autoimmune panel were unremarkable. Alpha-1 antitrypsin and ceruloplasmin levels were within normal limits. Bile sludge was seen on ultrasound. Magnetic resonance cholangiopancreatography (MRCP) abdomen showed segmental narrowing of the intrahepatic ducts. Endoscopic retrograde cholangiopancreatography (ERCP) was unremarkable. Liver biopsy showed mixed portal hepatitis, cholestasis, and biliary reactive changes with ceroid-loaded macrophages; a picture consistent with DILI. The patient was treated supportively and discharged with scheduled hepatology follow-up. At the one-month follow-up, his total bilirubin had fallen from a peak of 530 mol/L to 188 mol/L. The diagnosis of DILI can be made based on the timing of exposure and the exclusion of other etiologies. Liver enzymes normalized three to 12 months after product discontinuation. We hope this report will remind primary care physicians of the potential hepatotoxic side effects of muscle-building compounds and encourage them to report suspected DILI to the FDA using the MedWatch system.

## Introduction

Drug-induced liver injury (DILI) accounts for around 13% of acute liver failure (ALF) cases in the United States [1]. The probability of a drug causing hepatic damage ranges from one in 10,000 to one in 100,000 [2]. Though most cases of DILI tend to be benign with improvement after drug discontinuation, DILI remains the leading cause of death from acute liver failure [3]. DILI is categorized as either predictable (direct hepatoxicity from intrinsically hepatotoxic agents) or unpredictable (idiosyncratic), with the latter being more predominant [3]. A third type, called indirect DILI, is more likely due to liver injury from the drug’s actions rather than its hepatotoxic effect [4]. Early recognition and cessation of the causative drug are imperative to prevent the development of acute liver failure or chronic liver disease. Selective androgen receptor modulators (SARMs) were developed to increase muscle mass while avoiding the side effects of conventional androgenic steroids [2]. Though SARMs are not approved by the US Food and Drug Administration (FDA), they are widely available and marketed online to enhance athletic performance. We are presenting a case report of a young man who developed jaundice and transaminitis after a four-month course of RAD-140, an investigational SARM.

## Case presentation

A 22-year-old male patient, with a past medical history of acid reflux controlled on proton pump inhibitors, presented with acute, progressively worsening jaundice for one to two weeks. He reported taking RAD-140 for four months prior to the presentation. Subsequently, he developed nausea and generalized fatigue, dark urine, acholic light grey-colored stool, associated with pruritus and jaundice. In addition, he also used a performance-enhancing supplement called "buck upper workout" simultaneously. He denied prior history of hepatitis, substance drug use, sick contacts, recent travel, significant current alcohol intake, or previous alcohol use disorder.

The patient was hemodynamically stable on presentation. Scleral icterus was noted on the examination. Complete blood count parameters were within range. Liver panel showed alkaline phosphatase (ALP) 5.3 µkat/L, alanine transaminase (ALT) 1.66 µkat/L, aspartate transaminase (AST) 1.18 µkat/L, direct bilirubin 294 µmol/L, total bilirubin 427.5 µmol/L, and international normalized ratio (INR) 0.9. Viral workups including hepatitis A, hepatitis B, hepatitis C, herpes simplex, Epstein-Barr, and cytomegalovirus were negative. Alpha-1 antitrypsin and ceruloplasmin levels were within normal limits. Anti-nuclear antibody (ANA) was positive with a titer of 1:160. Anti-smooth muscle antibodies (ASMA) and anti-mitochondrial antibodies (AMA) were within normal limits. HIV, gonorrhea, and Chlamydia screening were negative as well.

**Figure 1:**
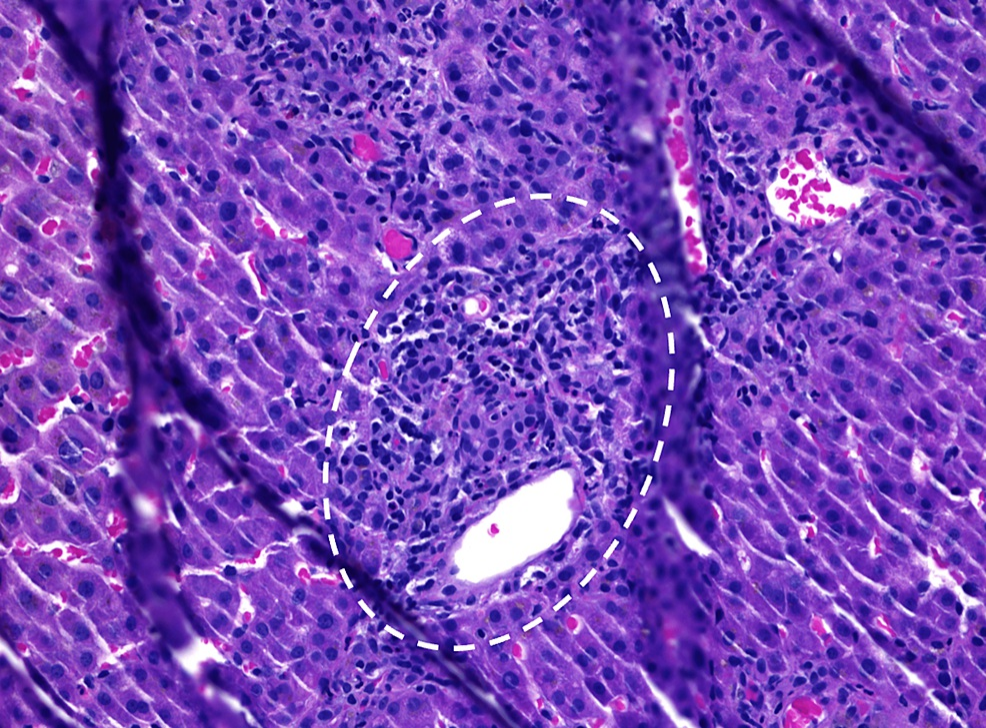
Liver biopsy (hematoxylin and eosin, 20x). The image is showing lymphocytic infiltration within the portal area (encircled).

**Figure 2:**
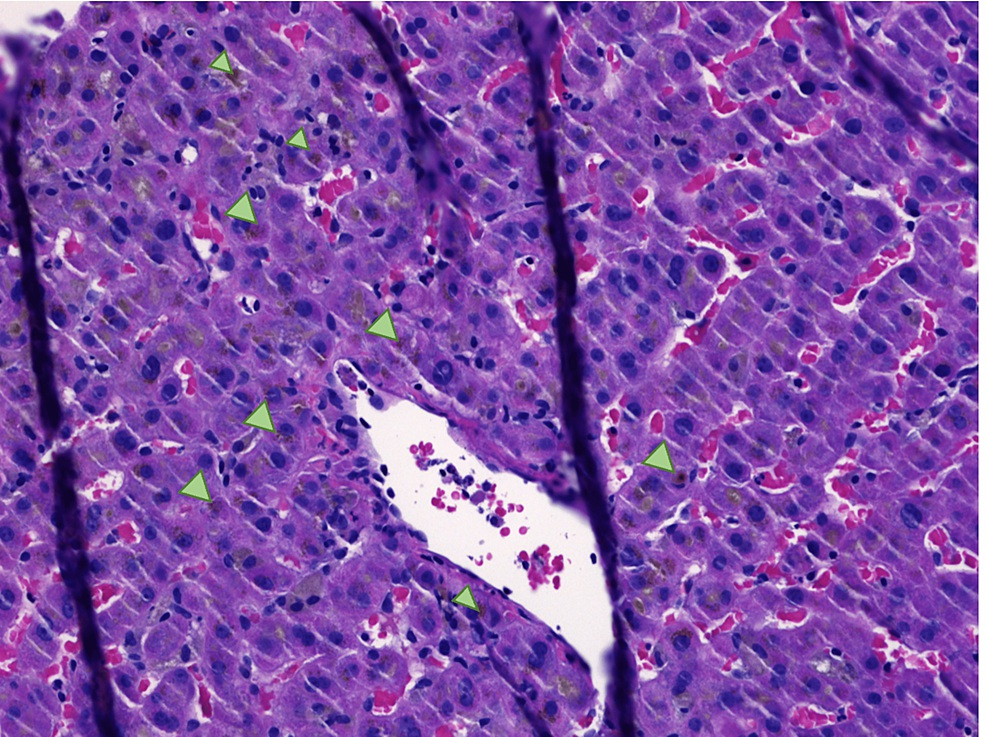
Liver biopsy (hematoxylin and eosin, 20x). The image is showing lobular cholestasis (arrowheads).

**Figure 3:**
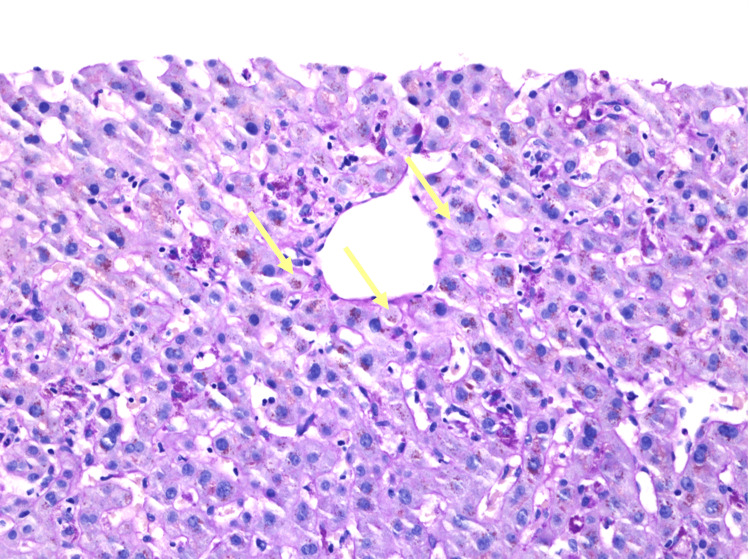
Liver biopsy (hematoxylin and eosin, 20x). The image is showing ceroid-laden macrophages (arrows).

Right upper quadrant ultrasound showed biliary sludge. Magnetic resonance cholangiopancreatography (MRCP) of the abdomen revealed mild segmental narrowing involving intrahepatic ducts above the level of the common hepatic confluence. Endoscopic retrograde cholangiopancreatography (ERCP) was unrevealing, with a normal cholangiogram, no evidence of primary sclerosing cholangitis (PSC) changes, and no stricture or narrowing. Liver biopsy showed mixed portal hepatitis (Figure 1), cholestasis (Figure 2), and biliary reactive changes with numerous ceroid-laden macrophages (Figure 3). The specimen stained negative for steatosis, iron, copper, Periodic acid-Schiff with diastase (PAS-D) globule, or pathologic fibrosis (Figures 1-3).

After excluding other etiologies, the bland cholestasis was considered consistent with drug-induced liver injury secondary to SARMs. Supportive measures were initiated, including hydration, clear liquid diet, anti-emetics, and an anti-pruritic. The patient was discharged home on hospital day three with a prescription of cholestyramine for pruritus.

**Figure 4:**
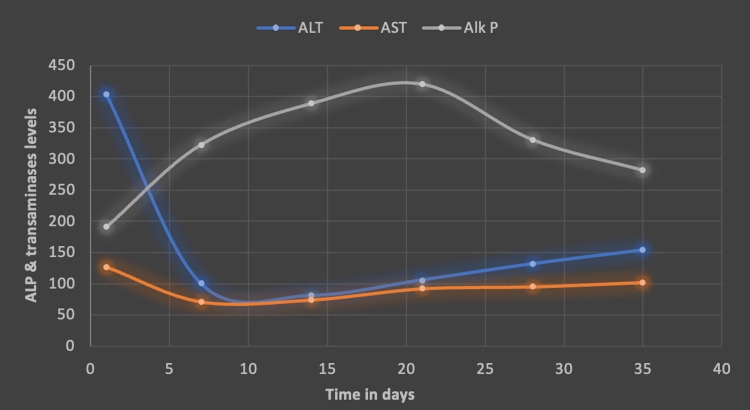
Alkaline phosphatase (ALP) and transaminases trend. Graph showing ALP and transaminases trend over 35 days. AST: aspartate transaminase; Alk P: alkaline phosphatase

**Figure 5:**
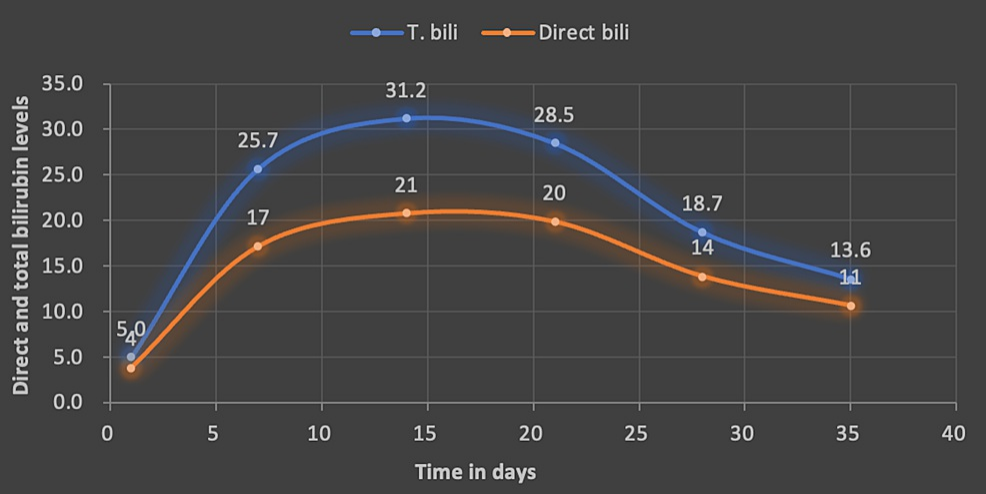
Direct and total bilirubin trend. Graph showing direct and total bilirubin trend. We notice that total bilirubin peaked at 31 and down trended to 11 at 35 days. T. bill: total bilirubin

He was seen in a follow-up outpatient appointment after one month, where he reported cessation of SARM use. Liver enzymes showed improvement (Figure 4). Total bilirubin, which had peaked at 31, down trended to 11 at day 35 (Figure 5). He reported that his symptoms had subsided, though he still had jaundice and mild fatigue. The patient was advised to exercise moderately and maintain adequate hydration, along with avoidance of herbal and hormonal supplements. He was scheduled to follow-up with the hepatology clinic periodically till his liver enzymes normalized. A full recovery was expected.

## Discussion

Drug-induced liver injury is an adverse drug reaction characterized by elevated liver enzymes, sometimes leading to ALF [5]. It is a well-known but rare diagnosis of exclusion. Among prescription drugs, antibiotics are the most common class of drugs that cause DILI. Herbal and dietary supplements (HDS) are the second most common causes [5]. Polymorphism of hepatic transporter proteins is a known predisposing factor [6]. Deciphering genetic variations and adaptive immunity with human leukocyte antigen (HLA) typing and single nucleotide polymorphism (SNP) evaluation are central to understanding DILI [6].

Clinically significant DILI has been defined as one of the following: (1) AST >5x upper limit of normal (ULN) or ALP >2x ULN on two separate occasions at least 24 hours apart; (2) total serum bilirubin >42.74 umol/L with elevated AST, ALT, or ALP; or (3) INR >1.5 with elevated serum AST, ALT, or ALP [5,7,8].

The R-value is defined as serum ALT/ULN divided by serum ALP/ULN and is best calculated at first presentation [9]. It helps classify the type of liver injury; >5 indicates hepatocellular injury, <2 suggests cholestatic injury, whereas R-value between 2 and 5 signifies a mixed injury pattern [9]. Our patient had an R-score of 1.5, indicating a cholestatic liver injury.

Causality assessment scores that aid in diagnosis include the Roussel-Uclaf Causality Assessment Method (RUCAM) scale, the Maria and Victorino System, clinical and diagnostic scale (CDS), and drug-induced liver injury network (DILIN) [10]. The RUCAM score is most widely used to determine the likelihood of the drug causing liver injury. It considers the phenotype, latency, de-challenge duration, and likelihood, based on the known potential hepatotoxicity of the agent. The final score ranges from 0, which rules out the drug as the cause of liver damage, to >8, which suggests that the drug is a "highly probable" cause. Our patient was found to have a RUCAM score of 4, which is interpreted as a "probable" cause of DILI [10].

Other alternative causes of liver injury must be ruled out. Causes include viral hepatitis, Wilson’s disease in younger patients, primary biliary cholangitis, and alcoholic hepatitis. Autoimmune hepatitis is always on top of the differential diagnosis list in all DILI cases. Drugs like nitrofurantoin and minocycline are well-known to have a high tendency to trigger an autoimmune-like DILI with high ANA and ASMA titers [11]. Our patient solely had a positive ANA titer in the setting of negative other autoimmune hepatitis (AIH) markers, which pointed towards the need for a liver biopsy to rule out drug-induced AIH.

“Direct” hepatocyte injury is the most common form and is dose-dependent and predictable [3]. It includes acute hepatic necrosis, sinusoidal obstruction syndrome (hepatic veno-occlusive disease), nodular regenerative hyperplasia, and microvesicular steatosis [12]. The “Idiosyncratic” type, on the other hand, is not dose-dependent and is unpredictable. It arises due to relatively unknown mechanisms. Indirect hepatotoxicity can manifest as fatty liver, acute hepatitis, or immune-mediated injury [13]. Recently released practice guidance by the American Association for the Study of Liver Disease (AASLD) suggests that liver biopsy may not be needed for mild or self-remitting cases, though it can be useful in those with prolonged disease or uncertain diagnosis [14]. Histological eosinophilia and granulomas are associated with lower mortality, whereas hepatic necrosis, fibrosis, and portal venopathy are some findings associated with higher rates of liver failure potentially requiring transplantation [15].

General supportive therapy with antipyretics, antiemetics, analgesics, and hydration remains the mainstay of treatment [14]. A prospective longitudinal study of 1257 subjects found that 10% of patients required liver transplantation, 17% had chronic liver injury, while the remainder recovered without any long-term sequelae [8]. Those with pre-existing liver disease were found to have more severe DILI and higher mortality risk [8].

SARMs are non-steroidal, orally available synthetic compounds with tissue-selective activity on the androgen receptors in muscle and bone (with reduced activity in other tissues such as the prostate, seminal vesicles) [16]. They offer the advantage of reaping the beneficial effects of androgens in target tissues and reducing the unintended side effects elsewhere [16].

They have been proposed to treat osteoporosis, breast cancer, and muscle wasting [16]. They are not currently FDA-approved for human use. A warning was issued in 2017 raising serious safety concerns, including the potential to increase the risk of heart attack or stroke and liver damage [17]. Surprisingly, they are widely sold online as dietary supplements and are frequently used for muscle-building and enhancing athletic performance. Van Wagoner et al. studied the purity of such substances in 2016 [18]. They studied 44 products that were sold under the label of selective androgen receptor modulators. Twenty-three products (52%) contained one or more selective androgen receptor modulators - including Ostarine, LGD-4033, or andarine. Seventeen products (39%) contained other unapproved drugs like ibutamoren (growth hormone secretagogue), GW501516 (peroxisome proliferator-activated receptor-δ agonist), and SR9009 (Rev-ErbA agonist) [18].

Some of the known SARMs included enobosarm (Ostarine or MK-2866 or S-22), andarine (S4), ligandrol (LGD-4033), and testolone (RAD-140) [16]. The drug-induced liver injury in the case of SARMs is thought to be idiosyncratic, extrapolating a similar mechanism with androgenic steroids [16].

A review of the existing literature revealed only four published reports of SARM-DILI, covering six cases (Table 1) [19-22]. All were published in and after 2020. All cases involved males between the ages of 19 and 40 years. The agents used included ligandrol (n=4), Ostarine (n=2), and RAD-140 (n=1). Though all patients presented with jaundice, there was variability in symptom onset from agent cessation. All cases had no history of liver disease. All but one of them reported an alcohol use disorder. Peak time of liver enzyme elevation varied. Most of the previously documented cases were cholestatic (n=3), rather than mixed (n=2) or hepatocellular (n=1). The current case also showed a mixed pattern. A cholestatic picture was obtained consistently on histology, including our case. Ursodiol has been used for treatment in a few cases [3]. The documented time to normalization of liver enzymes varied from three to 12 months, further highlighting the idiosyncratic nature of DILI.

**Table 1:** Summary of published case reports on selective androgen receptor modulators (SARMs)-induced liver injury. *Converted value. Reference ranges are included in parenthesis wherever available; all parameters are in SI units. Bi: bilirubin; ALT: alanine aminotransferase; AST: aspartate aminotransferase; ALP: alkaline phosphatase; GGT: gamma-glutamyl transferase; PC: platelet count; INR: international normalized ratio; NR: not reported

Author	Age and Sex	Presentation	Agent (duration)	Alcohol use	Bilirubin (Bi) presentation > Bi peak	ALT presentation > ALT peak	AST presentation > AST peak	ALP presentation > ALP peak	GGT on presentation	PC on presentation	INR on presentation	R-ratio	RUCAM score	Imaging	Histology	Treatment	Prognosis
Flores et al. in 2020 [19]	24-year-old male	Jaundice, anorexia, nausea, lethargy, 5 kg weight loss for 5 weeks; 1 week after cessation of agent	Ligandrol (9 weeks)	Binge drinking once a month	116 µmol/L (2-20) > 116 µmol/L at week 0	4.53 µkat/L*(<0.66*) > 9.78 at 2 weeks	1.84 µkat/L*(<0.66*) > 2.91 µkat/L* at 2 weeks	4.82 µkat/L* (0.5-1.83*) > 4.82 µkat/L*	1.03 µkat/L*(<1.83)	387 x 10^9 ^(150-450)	1.0	8.22	7	Liver US - no biliary obstruction	NR	NR	4 months for enzymes to return to baseline
Flores et al. in 2020 [19]	49-year-old male	Jaundice and itching for 5 weeks	RAD-140 (intermittently for 4 weeks, 4 months)	Insignificant	291 µmol/L (2-20) > 346 µmol/L at 3 weeks,	0.86 µkat/L*(<0.66*) > 2.86 µkat/L* at 8 weeks	0.98 µkat/L*(<0.66*) > 2.04 µkat/L* at 8 weeks	5.45 µkat/L*(0.5-1.83*) > 5.45 µkat/L* at week 0	NR	347 x 10^9 ^(150-450 x 10^9^)	1.2	5.0	6	Liver imaging - no biliary obstruction	Moderate cholestasis with ductopenia and minimal fibrosis and inflammation	Ursodiol, cholestyramine	12 months for enzymes to return to baseline
Barbara et al. in 2020 [20]	32-year-old-male	Fatigue, pruritus, and weight loss for 50 days	Ligandrol 10 mg daily (2 weeks)	NR	41 µmol/L* > 653.22 µmol/L*at Day 46	3.8 µkat/L* > 4.05 µkat/L* on day 26	91 IU/L > 1.51 µkat/L* on day 22	.47 µkat/L*>0.75 µkat/L* at day 54	NR	NR	1.1	NR	NR	Abdominal US and CT - hepatomegaly; MRCP - small hepatic cyst, splenomegaly, no biliary dilatation	Cholestatic hepatitis with mild portal, periportal, and perisinusoidal fibrosis; no hyaline globules on PAS diastase stain, 2+ irons staining of hepatocytes	NR	NR
Koller et al. in 2021 [21]	19-year-old-male	Dark urine, yellow sclera, thin light-colored stools, after 3 weeks of PCT	Ligandrol once capsule daily (4 weeks, 3 weeks of post-cycle therapy)	None	238 µmol/L (3.4-17.1) > NR	ALT 2.2 µkat/L (0.2-0.80) > NR	NR	ALP 1.54 µkat/L (0.67-2.15) > NR	0.41 µkat/L (0.18- 1.02)	NR	NR	3.9	6	Abdominal US - normal liver and spleen size, no duct dilation	Mild septal fibrosis, and canalicular cholestasis in the hepatocytes with numerous biliary plugs. Few necrotic hepatocytes, centrilobular mostly lymphocytic infiltrate, and ductopenia.	1000 mg ursodiol daily for 2 months	3 months for enzymes to return to baseline
Koller et al. in 2021 [21]	28-year-old-male	Nausea, fatigue, and jaundice, after 4th dose of PCT	Ligandrol (3 months), and Ostarine (3 weeks later )	None	401 µmol/L > NR	2.4 µkat/L > NR	NR	1.54 µkat/L > NR	NR	NR	NR	3.3	NR	MRI - hepatomegaly without biliary pathology	Mild bridging fibrosis, destruction of bile ducts, centrilobular canalicular cholestasis with numerous bile plugs in the canaliculi, phagocytosis hepatocellular apoptosis, and perivenular necrosis	300 mg intravenous N-acetyl cysteine 4 times daily, 1000 mg oral ursodiol daily, and 450 mg silymarin daily	NR
Bedi et al. in 2021 [22]	Early 40s male	Jaundice, anorexia, weight loss, lethargy, diarrhea	Ostarine (2 months)	None	0.34 µmol/L > 0.735 µmol/L at 4 weeks	1.86 µkat/L* > NR	1.15 µkat/L* > NR	268 U/L > NR	49 U/L	313x 10^9^	1.3	0.8	NR	US and CT - negative for duct dilatation, cirrhosis, hepatomegaly, and intraabdominal VTE; MRCP - normal hepatic and biliary anatomy	Mild bile ductular reaction with very mild duct damage and minimal inflammation; moderate to severe cholestasis	NR	Gradual improvement over several months
This study	20-year-old-male	Dark urine, light stools, pruritus	RAD-140 (4 months)	None	427.5 µmol/L* (5.13-20.52) > 533.5 µmol/L* at 7 days	1.66 µkat/L* (0-0.56*) > 2.56 µkat/L* at 28 days	1.18 µkat/*L (0-0.56*) > 1.69 µkat/L* at 28 days	5.38 µkat/L* (0.73-2*) > 7 µkat/L* at 14 days	NR	NR	0.9	1.5	4	US-biliary sludge; MRCP - mild segmental narrowing involving intrahepatic ducts; ERCP-normal cholangiogram, no signs of PSC, no strictures/narrowing	Mixed portal hepatitis, cholestasis, and biliary reactive changes, numerous ceroid-laden macrophages	Supportive measures	Bi improved from 530 to 188 at 4 weeks after follow-up, clinical progression is still being monitored.

The SARM properties of RAD-140 were first described by Siegel et al. in 2022 [23]. It was studied in preclinical rat models to determine its properties, including oral efficacy. The dose required to increase muscle weight was less than that required to stimulate prostate weight gain. Surprisingly, there was little effect on the liver enzymes even at a dose 10-fold greater than the effective dose. Its role has been further investigated in estrogen receptor-positive breast cancer and cachexia models [24,25].

Our patient consumed RAD-140 for four months before developing gastrointestinal symptoms. After ruling out other known causes of acute liver injury, the diagnosis was made based on the timing and improvement in symptoms with cessation of the offending agent. Liver biopsy showed mixed portal hepatitis with cholestasis and biliary reactive changes. These changes were consistently observed in SARM-induced liver injury, as described above. A toxicological analysis could have helped identify other contaminants, which could have additionally contributed to hepatic insult.

## Conclusions

More patient education by primary care physicians about the potential hepatotoxic side effects of muscle-building substances (not approved by the FDA) may help reduce the number of future DILI cases secondary to SARMs. We hope this case will increase clinicians' awareness of this entity and encourage them to voluntarily report suspected DILI to the FDA by using the MedWatch System. 
